# In this month’s *Bulletin*

**DOI:** 10.2471/BLT.15.000615

**Published:** 2015-06-01

In an editorial, Amina Aitsi-Selmi & Virgina Murray (362) introduce the *Sendai framework for disaster risk reduction*.

In the news section, Patrick Adams and Fiona Fleck report on efforts to provide health information in languages other than English (365–366). Jeremy Farrar talks to Fiona Fleck about the Wellcome Trust’s priorities for research funding (367–368).

## Rwanda

### Filling supply gaps

Agnes Binagwaho et al. (429–434) describe efforts to coordinate eye-care services nationwide.

## South Africa

### Monitoring the care given to babies

Emma R Allanson & Robert C Pattinson (424–428) study the associations between quality-of-care audits and perinatal mortality.

## China

### Doctors in megacities

Xiaolin Wei et al. (407–416) measure patients’ perceptions of primary care in Shanghai and Shenzhen.

## United Republic of Tanzania

### Improving maternal and newborn health in rural areas

Ulrika Baker et al. (380–389) track implementation bottlenecks.

## 21 European countries

### Impacts of reduced public health expenditure

Aaron Reeves et al. (369–379) model the effects of economic recession on tuberculosis control.

## Georgia 

### Two hits to the lungs

Medea Gegia et al. (390–399) track the influence of smoking on tuberculosis treatment outcomes. 

## Australia 

### Early deaths of burn survivors

Janine M Duke et al. (400–406) compare long-term outcomes of burn survivors to the non-injured adult population. 

## Global

### Climate change and infectious diseases

Ryota Sakamoto (435–436) argues that legionellosis is one of the diseases influenced by climate.

### Three fatal delays

Emilie J Calvello et al. (417–423) apply lessons learnt from obstetric emergencies. 

